# Intracranial efficacy of alectinib in ALK-positive NSCLC patients with CNS metastases—a multicenter retrospective study

**DOI:** 10.1186/s12916-021-02207-x

**Published:** 2022-01-18

**Authors:** Zihua Zou, Puyuan Xing, Xuezhi Hao, Yan Wang, Xia Song, Li Shan, Cuiying Zhang, Ziling Liu, Kewei Ma, Guilan Dong, Junling Li

**Affiliations:** 1 Department of Medical Oncology, National Cancer Center/National Clinical Research Center for Cancer/Cancer Hospital, Chinese Academy of Medical Sciences and Peking Union Medical College, Beijing, People’s Republic of China; 2 Department of Respiratory Medicine, Shanxi Provincial Cancer Hospital, Taiyuan, People’s Republic of China; 3 Department of Thoracic oncology, Tumor Hospital Affiliated to Xinjiang Medical University, Urumqi, People’s Republic of China; 4 Cancer center, Inner Mongolia Autonomous Region People’s Hospita, Huhhot, People’s Republic of China; 5 Cancer center, The First Hospital of Jilin University, Changchun, People’s Republic of China; 6 Department of Medical Oncology, Tangshan People’s Hospital, Tangshan, People’s Republic of China

**Keywords:** ALK-positive non-small cell lung cancer, Central nervous system metastases, Brain metastases, Leptomeningeal metastases, Alectinib

## Abstract

**Background:**

Central nervous system (CNS) metastases in patients with ALK-positive non-small cell lung cancer (NSCLC) are a cause of substantial morbidity and mortality. Although alectinib had demonstrated promising intracranial efficacy in several clinical trials, data were limited on its CNS activity in real-world settings.

**Methods:**

In this retrospective study, ALK-positive NSCLC patients with brain metastases (BM) or leptomeningeal metastases (LM) from six hospitals in China were divided into three cohorts based on the treatment history before the administration of alectinib. ALK-TKI-naive patients were enrolled in cohort 1, cohort 2 included patients who experienced intracranial progression with or without extracranial progression after treatment with crizotinib, and cohort 3 included patients who developed progression only in CNS following treatment with other second-generation ALK-TKIs. The definition and evaluation of intracranial and extracranial lesions were based on Response Evaluation Criteria in Solid Tumors version 1.1.

**Results:**

Sixty-five patients were eligible and included in our study (cohort 1: 20, cohort 2: 32, cohort 3: 13). For the overall population and patients with uncontrolled CNS metastases, similar intracranial response in CNS target lesions was observed: cohort 1: 81.8% and 80%; cohort 2: 76.5% and 86.7%; cohort 3: 42.8% and 33.3%. For patients in these three cohorts, 75% (6/8), 78.6% (11/14), and 83.3% (5/6) were reported to have significant improvement in CNS-related symptoms respectively. The number of patients who were in need of mannitol or corticosteroids decreased remarkably after the treatment of alectinib (*p* < 0.001), and there was also a steep fall-over in the number of patients with ECOG ≥2 points before and after the administration of alectinib (*p* = 0.003). All patients (8/8) diagnosed with LM ± BM experienced substantial alleviation in CNS-related symptoms. In cohort 1 and cohort 2, no significant difference in CNS-time to progression was found between patients with symptomatic or asymptomatic BM when treated with alectinib alone.

**Conclusions:**

Our study substantiated the potent CNS activity of alectinib in real-world settings. Patients with symptomatic and asymptomatic BM could benefit from alectinib comparatively, which indicated that alectinib alone might defer the timing of local treatment. However, our results should be treated cautiously owing to limited sample size.

**Supplementary Information:**

The online version contains supplementary material available at 10.1186/s12916-021-02207-x.

## Background

In the era of chemotherapy, patients with advanced non-small cell lung cancer (NSCLC) have experienced dismal prognoses. Over the past few decades, there has been huge progress in tumor molecular biology. Several driver gene mutations such as EGFR, ALK, and ROS1 have been found, resulting in a dramatic change in the treatment landscape of non-squamous NSCLC: from empirical cytotoxic drugs to targeted therapy. Currently, long-term survival for patients with driver gene mutations has been significantly improved with the help of tyrosine kinase inhibitors (TKIs). ALK gene rearrangement has been considered as “diamond mutation”: prior studies revealed that patients with advanced ALK+NSCLC could live approximately 7 years after sequential treatment of multiple generations of ALK-TKIs and closed multidisciplinary collaborations [1]. However, metastases in the central nervous system (CNS) (including brain metastases [BM] and leptomeningeal metastases [LM]; BM usually indicates metastases in brain parenchyma) still pose great threats to quality of life (QoL), neurological cognitive functions, and survival for patients with advanced NSCLC. About 30–40% of patients [1, 2] with advanced ALK+NSCLC have CNS metastases at the time of initial diagnosis, and roughly 50–60% of patients experience CNS metastases following the treatment of first-generation ALK-TKI (crizotinib) [3–6]. Given the high incidence rate of CNS metastases in advanced ALK+NSCLC patients, an optimal treatment strategy for CNS metastases is desperately needed.

Alectinib, a second-generation ALK-TKI, which is not the substrate of p-glycoprotein, enjoys an extremely high penetration rate across the blood–brain barrier (BBB) [7]. In a pooled analysis of two phase II studies, alectinib demonstrated an intracranial objective response rate (ic-ORR) over 60% in crizotinib-resistant patients with measurable CNS metastases [8]. A robust CNS activity of alectinib had also been observed in the phase III ALEX trial, with ic-ORR over 75% for TKI-naive patients with measurable BM [9].

Although CNS efficacy of alectinib had been firmly confirmed in several clinical trials, it should be noted that patients with symptomatic or unstable CNS metastases were excluded in all clinical trials of alectinib [4–6, 10, 11]. Thus, there had been limited data about the intracranial efficacy of alectinib in these patients. Up to now, surgical resection, stereotactic radiosurgery (SRS), and whole brain radiotherapy (WBRT) have been the mainstream strategies for symptomatic or unstable CNS metastases; nonetheless, these treatment options may cause radio-necrosis (RN) and impairment in cognitive function, with some researchers even reporting that ALK+NSCLC patients were especially prone to develop RN (HR 6.36, *p* < 0.001) [12–14]. Whether alectinib can delay or reduce the need for local treatment for patients with symptomatic or unstable CNS metastases is yet to be fully investigated. Additionally, previous research showed a higher penetration rate across the BBB of alectinib compared with crizotinib and ceritinib. It also remains to be seen whether alectinib can achieve further inhibition in intracranial lesions for patients who experience progression only in CNS following the treatment of other second-generation ALK-TKIs (ceritinib, CT707, or WX-0593).

Therefore, we conducted this multicenter retrospective analysis in China to explore the CNS activity of alectinib in a real-world setting.

## Methods

### Patients and data collection

Patients diagnosed with advanced ALK+NSCLC who had baseline CNS metastases before the administration of alectinib were included from six hospitals in China from 2017 to 2020. MRI scans for intracranial lesions and CT scans for extracranial lesions at baseline and during the follow-up period were required; symptoms caused by BM were not mandatory, and patients could have received no or one or more prior ALK-TKIs before the initiation of alectinib; and prior CNS radiotherapy or surgery was allowed if the aforementioned criteria were met. Patients included in this study were divided into three cohorts based on the treatment history before the initiation of alectinib. ALK-TKI-naive patients were enrolled in cohort 1; cohort 2 included patients who experienced intracranial progression with or without extracranial progression after treatment with crizotinib; and patients who developed progression only in CNS following treatment with other second-generation ALK inhibitors (ceritinib, CT707, WX-0593) were classified into cohort 3. The data cutoff date was June 1, 2021.

### Assessments

The definition and evaluation of intracranial or extracranial lesions were based on the Response Evaluation Criteria in Solid Tumors version 1.1 (RECIST 1.1). In other words, up to five target lesions (≥ 1 cm) in the whole body and up to two target lesions (≥ 1 cm) in each organ were included; ic-ORR, intracranial disease control rate, extracranial objective response rate (ex-ORR), and extracranial disease control rate were recorded; when taking intracranial and extracranial lesions together, overall objective response rate (o-ORR) and overall disease control rate were recorded; tumor shrinkage rate in CNS target lesions was also analyzed. The extent of improvement in CNS-related symptoms was mainly based on the subjective report from patients, which could be categorized into four different levels (significant improvement, moderate improvement, no improvement, deterioration); performance status and the proportions of patients who were in need of mannitol or corticosteroids before or after the initiation of alectinib were also recorded. Intracranial oligo-progression was defined as progression in one to three brain lesions, while patients who developed progression in more than three BMs were deemed as having intracranial multi-progression. Radiological assessment was obtained at baseline and then every 1 to 3 months.

### Statistical analysis

Statistical analysis was conducted using the SPSS 26.0 statistical software (SPSS, Inc., Chicago, IL, USA). The distribution of patients and baseline demographic/clinical characteristics were described using frequency analysis. Objective response rate or disease control rate was estimated with 95% confidence interval (CI) based on the exact binomial distribution. Intracranial duration of response (ic-DOR) was defined as the time from the first CNS response (complete response [CR] + partial response [PR] in CNS target lesions and CR in CNS nontarget lesions) until CNS progression. CNS time to progression (CNS TTP) was calculated from the start date of administration of alectinib until CNS progression. Progression-free survival (PFS) was calculated from the start date of alectinib until progression or any death event. Overall survival (OS) was calculated from the start date of alectinib to any death event. Differences among groups were compared using Fisher exact test for categorical data and *t*-tests for continuous data. The survival curves were estimated using the Kaplan-Meier method, while differences in the variables were calculated using the log-rank test. A two-sided *p* value < 0.5 was considered statistically significant.

## Results

### Patients’ characteristics

From July 2017 to September 2020, 65 patients (cohort 1: *n* = 20, cohort 2: *n* = 30, cohort 3: *n* = 13) with baseline CNS metastases treated with alectinib from six hospitals in China were included in our study. Patients’ baseline characteristics are described in Table 1. In each cohort, 11, 17, and 7 patients were found to have CNS target lesions; meanwhile, the median sums of maximum diameter of CNS target lesions were 2.7 cm (range 1 cm, 5.3 cm), 2.4 cm (range 1.2 cm, 5.1 cm), and 1.7 cm (range 1 cm, 3.2 cm) respectively. In each cohort, 8 (40%), 14 (43.7%), and 6 (46.2%) patients, respectively, were reported to develop symptoms attributable to CNS metastases. Headache (*n* = 22), dizziness (*n* = 13), vomiting (*n* = 12), and fatigue (*n* = 12) were more common, while hemiplegia (*n* = 2), diplopia (*n* = 1), and tinnitus (*n* = 1) were less frequently reported in these patients. In total, nine patients were diagnosed with LM with or without BM, of whom eight were reported to experience CNS-related symptoms. A total of 13 patients received brain radiotherapy or brain surgery before the initiation of alectinib; however, most patients enrolled in our study had uncontrolled CNS metastases before treatment with alectinib (cohort 1: *n* = 19, cohort 2: *n* = 30, cohort 3: *n* = 12) (uncontrolled CNS metastases meant: CNS metastases were not treated before the administration of alectinib or progressed following prior targeted therapy or local treatment). Extracranial lesions were found in 12, 29, and 12 patients in each cohort, respectively.

**Table 1:** Baseline characteristics for three cohorts

	Cohort 1, ***n*** = 20	Cohort 2, ***n*** = 32	Cohort 3, ***n*** = 13
Age (median)	52 (range 30, 76)	51 (range 23, 69)	55 (range 40, 71)
Sex (%)			
Male Female	6 (30%) 14 (70%)	16 (50%) 16 (50%	9 (69.2%) 4 (30.8%)
ECOG (%)			
0–1 ≥ 2	14 (70%) 6 (30%)	22 (68.7%) 10 (31.3%)	9 (69.2%) 4 (30.8%)
Smoking status (%)			
Never smoker Current or former smoker	16 (80%) 4 (20%)	23 (71.9%) 9 (28.1%)	5 (38.5%) 8 (61.5%)
Pathology (%)			
Adenocarcinoma Other type	19 (95%) 1 (5%)	31 (96.9%) 1 (3.1%)	13 (100%) 0 (0%)
Stage (%)			
IV Recurrence after surgery or radical radiation	11 (55%) 9 (45%)	21 (65.6%) 11 (34.4%)	10 (76.9%) 3 (23.1%)
Previous brain radiotherapy or surgery (%)			
Yes No	5 (25%) 15 (75%)	6 (18.7%) 26 (81.3%)	2 (15.4%) 11 (84.6%)
Uncontrolled CNS metastases (%)			
Yes No	19 (95%) 1 (5%)	30 (93.7%) 2 (6.3%)	12 (92.3%) 1 (7.7%)
Leptomeningeal metastases (%)			
Yes No	4 (20%) 16 (80%)	2 (6.3%) 30 (93.7%)	3 (23.1%) 10 (76.9%)
With CNS target lesion (%) Without CNS target lesion (%)	11 (55%) 9 (45%)	17 (53.1%) 15 (46.9%)	7 (53.8%) 6 (46.2%)
Median sums of maximum diameter in CNS target lesion	2.7 cm (range 1 cm, 5.3 cm)	2.4 cm (range 1.2 cm, 5.1 cm)	1.7 cm (range 1 cm, 3.2 cm)
Symptoms related to CNS lesion (%)			
Yes No	8 (40%) 12 (60%)	14 (43.7%) 18 (56.3%)	6 (46.2%) 7 (53.8%)
With extracranial lesion (%)	12 (60%)	29 (90.6%)	12 (92.3%)
Without extracranial lesion (%)	8 (40%)	3 (9.4%)	1 (7.7%)
With extracranial target lesion (%)	11 (55%)	12 (37.5%)	6 (46.2%)
Without extracranial target lesion (%)	9 (45%)	20 (62.5%)	7 (53.8%)

### Intracranial efficacy in overall population

**Fig. 1:**
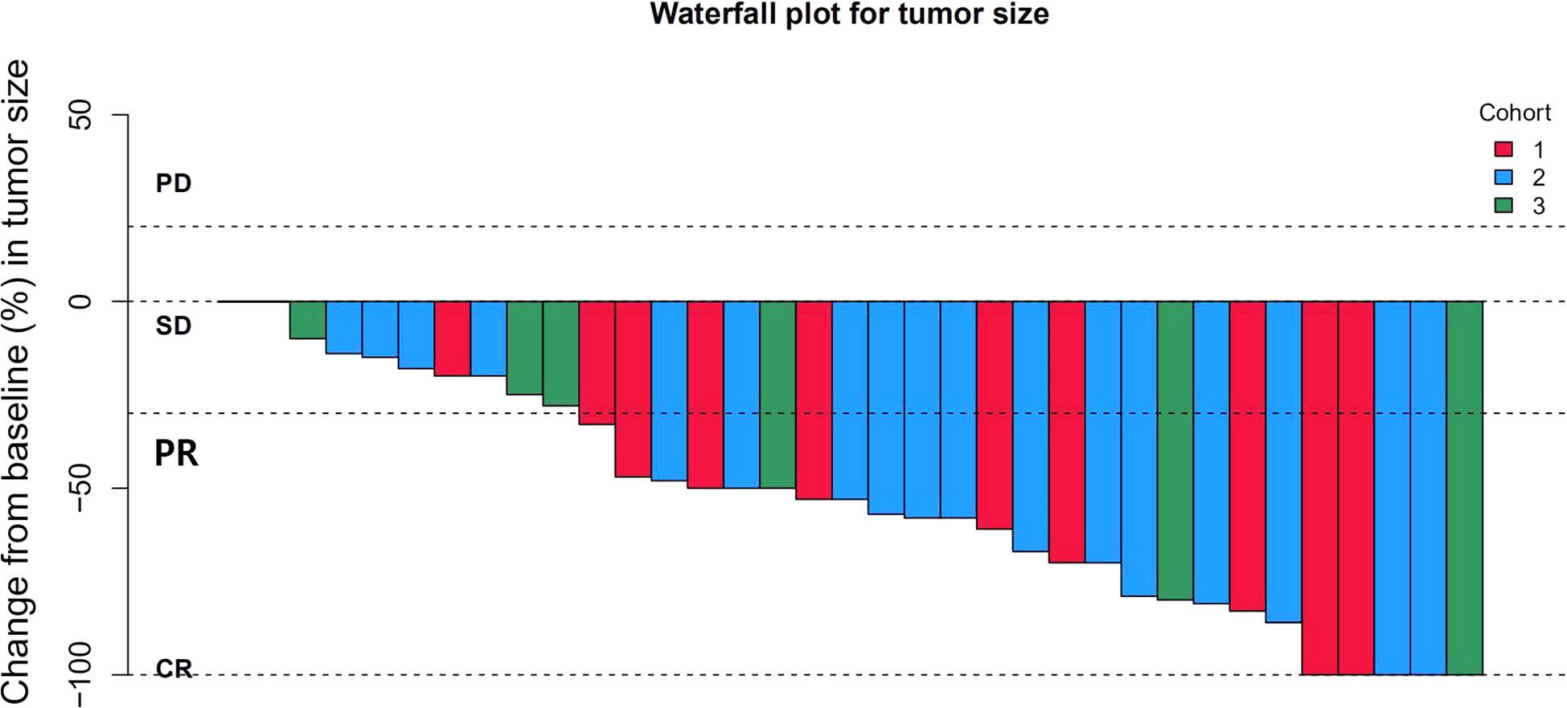
Tumor shrinkage rate in CNS target lesions; 11, 17, and 7 patients had CNS target lesions in these three cohorts respectively; median intracranial tumor shrinkage rate was 53% (range 0, 100%), 58% (range 14%, 100%), and 28% (range 0, 100%)

**Fig. 2:**
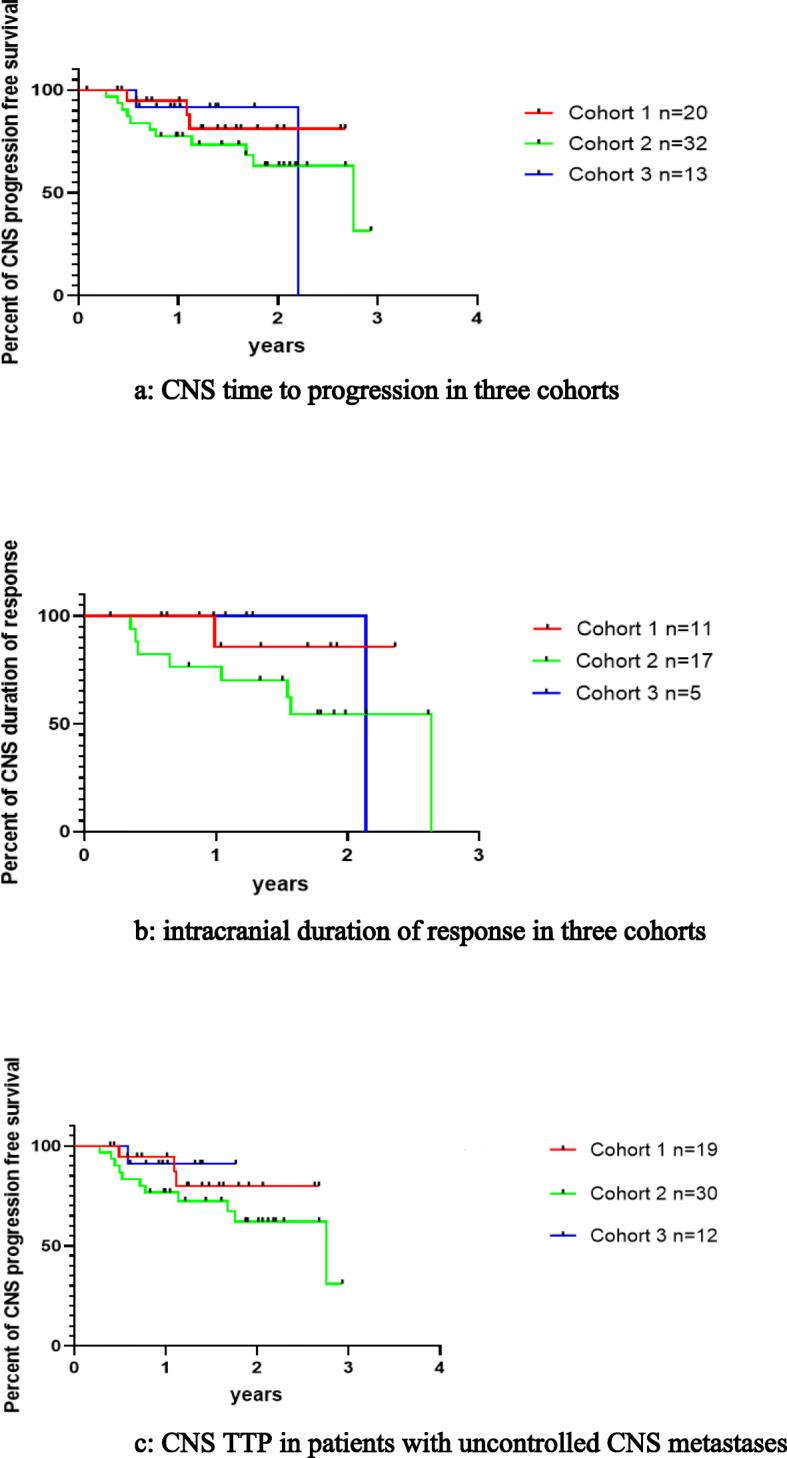
**a** CNS time to progression in three cohorts. With a median follow-up of 19.2 months, 22.5 months, and 15.8 months in these three cohorts respectively, CNS TTP was NE vs 33.0 m vs NE. **b** Intracranial duration of response in three cohorts. With median follow-up of 18.7 months, 22.7 months, and 16.8 months in these three cohorts respectively, ic-DOR was NE vs NE vs NE. **c** CNS TTP in patients with uncontrolled CNS metastases. With a median follow-up of 19.0 months, 22.6 months, and 12.3 months in these three cohorts respectively, CNS TTP was NE vs 33.0 m vs NE

**Fig. 3:**
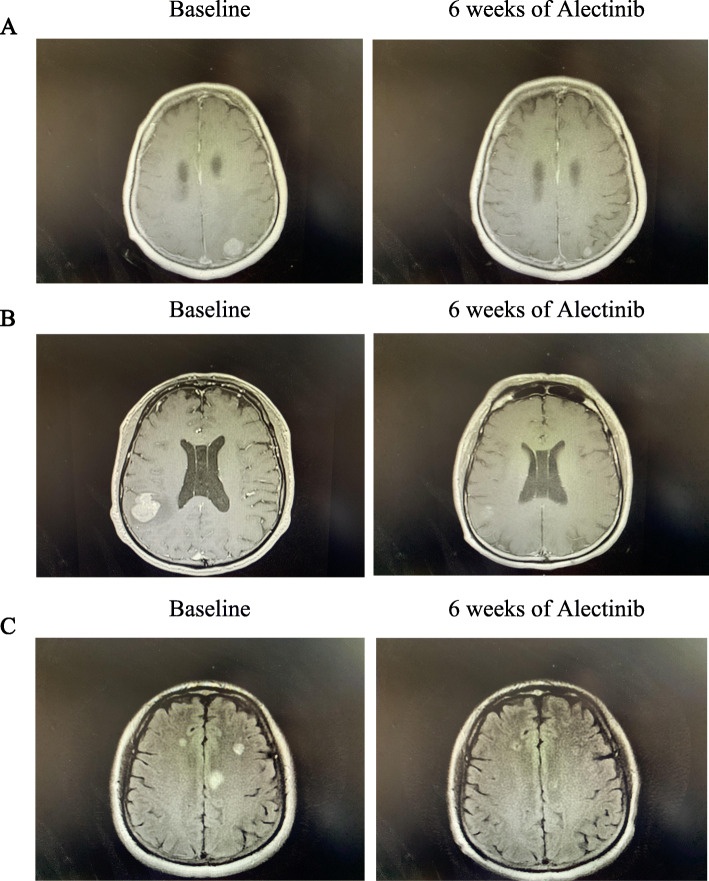
Typical examples in patients treated with alectinib. **A** Patient 1 who received first-line alectinib experienced significant alleviation in headache. **B** Headache and dizziness disappeared in patient 2 who received alectinib after the progression of crizotinib. **C** Headache and vomiting were largely improved in patient 3 who developed CNS progression following the treatment of ceritinib

The ic-ORR was 55% (4CR + 7PR), 53.1% (6CR + 11PR), and 38.5% (3CR + 2PR) in patients with or without CNS target lesions in each cohort, respectively (Table 2), and all patients reached disease control in CNS. In these three cohorts, 81.8% (2CR + 7PR), 76.5% (2CR + 11PR), and 42.8% (1CR + 2PR) of patients with CNS target lesions achieved CNS response, respectively, the median intracranial tumor shrinkage rate was 53% (range 0%, 100%), 58% (range 14%, 100%), 28% (range 0%, 100%) in these patients (Fig. 1). At the time of data cutoff, CNS TTP was NE, 33.0 m (95% CI: 15.8–50.2 m), and NE in these three cohorts separately, with a median follow-up of 19.2 months (95% CI: 17.7–20.8 m), 22.5 months (95% CI: 18.8–26.1 m), and 15.8 months (95% CI: 10.1–21.6 m), respectively (Fig. 2a). Furthermore, ic-DOR was NE in the three cohorts with median follow-up of 18.7 months (95% CI: 17.0–20.3 m), 22.7 months (95% CI: 20.5–25.0 m), and 16.8 months (95% CI: 16.1–17.4 m), respectively (Fig. 2b). Figure 3 demonstrates typical examples of radiological changes in patients with symptomatic BM.

**Table 2:** Intracranial efficacy of alectinib in three cohorts

	Cohort 1, ***n*** = 20	Cohort 2, ***n*** = 32	Cohort 3, ***n*** = 13
CNS ORR in all cohort patients (%)	55% [95% CI: 31.5–76.9%] (11/20)	53% [95% CI: 34.7–74.9%] (17/32)	38.5% [95% CI: 13.9–68.4%] (5/13)
CNS ORR in patients with CNS target lesions (%)	82% [95%CI:48.2%-97.7%] (9/11)	76.5% [95% CI: 50.1–93.2%] (13/17)	43% [95% CI: 9.9–81.6%] (3/7)
Median intracranial tumor shrinkage rate	53% Range 0, 100%	58% Range 14%, 100%	28% Range 0, 100%

### Intracranial efficacy in patients with uncontrolled CNS metastases

The ic-ORR was 52.6% (4CR + 6PR), 56.7% (6CR + 11PR), and 33.3% (2CR + 2PR) in each cohort and all patients achieved disease control in CNS. For patients with CNS target lesions in these three cohorts, 80% (2CR + 6PR), 86.7% (2CR + 11PR), and 33.3% (2PR) of them were reported to have CNS response; in the meantime, median intracranial tumor shrinkage rate was 55% (range 0%, 100%), 58% (range 14%, 100%), and 26% (range 0%, 80%) in these patients (Table 3). In total, 21 patients (21/25, 84%) experienced significant improvement in CNS-related symptoms following the treatment with alectinib, of whom 13 patients (13/17, 76.5%) had BM and 8 (8/8, 100%) had LM ± BM. With a median follow-up of 19.0 months (95% CI: 16.6–21.4 m), 22.6 months (95% CI: 20.4–24.8 m), and 12.3 months (95% CI: 5.0–19.6 m), CNS-TTP in patients with uncontrolled CNS metastases was NE, 33.0 months (95% CI: 15.7–50.3 m), NE (Fig. 2c).

**Table 3:** Intracranial efficacy of alectinib in patients with uncontrolled CNS metastases

	Cohort 1, ***n*** = 19	Cohort 2, ***n*** = 30	Cohort 3, ***n*** = 12
CNS ORR in patients with uncontrolled CNS metastases (%)	52.6% [95% CI: 28.9–75.6%] (10/19)	56.7% [95% CI: 37.4–74.5%] (17/30)	33.3% [95% CI: 9.9–65.1%] (4/12)
CNS ORR in patients with measurable uncontrolled CNS metastases (%)	80% [95% CI: 44.4–97.5%] (8/10)	86.7% [95% CI: 59.5–98.3%] (13/15)	33.3% [95% CI: 4.3–77.7%] (2/6)
Median intracranial tumor shrinkage rate	55% Range 0, 100%	58% Range 14%, 100%	26% Range 0, 80%

### Treatment outcomes in patients with symptomatic CNS metastases

Of 28 patients reported to have symptoms attributable to CNS metastases, 20 patients had symptomatic BM and 8 patients were diagnosed with symptomatic LM. Three patients who had received radiotherapy (RT) right before the initiation of alectinib were deemed to have controlled CNS metastases. Therefore, most patients (25/28) had uncontrolled CNS metastases before the administration of alectinib. For each cohort, 75% (6/8), 78.6% (11/14), and 83% (5/6) of patients experienced significant improvement in CNS-related symptoms (Table 4); the remaining proportion of these patients (2/8, 3/13, 1/6) was reported to have at least no deterioration in symptoms, and out of them, further salvage RT was only needed in two patients. Mannitol or corticosteroids were needed to alleviate symptoms in 18 patients before the initiation of alectinib, whereas only three patients were still in need of these drugs a half-month after the administration of alectinib; therefore, the number of patients who were in need of these drugs decreased remarkably following the treatment with alectinib (as was shown in the Fisher test *p* < 0.001) (Table 5). Over 70% of patients experienced improvement in ECOG by at least one point following the treatment with alectinib; furthermore, ECOG improved by two points was seen in one-fifth of patients with CNS-related symptoms (Table 6). Similarly, there was also a steep fall-over in the number of patients with ECOG ≥ 2 points before and after the administration of alectinib (as was shown in the Fisher test *p* = 0.003) (Table 7).

**Table 4:** Improvement in CNS-related symptoms

	Cohort 1, ***n*** = 20	Cohort 2, ***n*** = 32	Cohort 3, ***n*** = 13
Significant improvement in CNS-related symptoms (%)	75% (6/8)	78.6% (11/14)	83.3% (5/6)
Moderate improvement in CNS-related symptoms	25% (2/8)	7.1% (1/14)	16.7% (1/6)
No improvement in CNS-related symptoms	0	14.3% (2/14)	0
Deterioration in CNS-related symptoms	0	0	0

**Table 5:** Treatment of mannitol or corticosteroids before or after the administration of alectinib

Patients with CNS-related symptoms in the baseline, ***n*** = 28	Before the initiation of alectinib	After the treatment of alectinib
Number of patients who needed mannitol or corticosteroids	18 (64.3%)	3 (10.7%)
Number of patients who didn't need mannitol or corticosteroids	10 (35.7%)	25(89.3%)

Fisher exact test: *p* < 0.001, the number of patients who were in need of mannitol or corticosteroids decreased remarkably after the treatment of alectinib

**Table 6:** Improvement in ECOG after the treatment of alectinib

Patients with CNS-related symptoms in the baseline, ***n*** = 28	Number of patients
No improvement in ECOG after the treatment of alectinib	8 (28.6%)
ECOG was improved by 1 point after the treatment of alectinib	14 (50%)
ECOG was improved by 2 points after the treatment of alectinib	6 (21.4%)

**Table 7:** Performance status before or after the administration of alectinib

Patients with CNS-related symptoms in the baseline, ***n*** = 28	Before the initiation of alectinib	After the treatment of alectinib
ECOG 0-1	9 (32.1%)	21 (75%)
ECOG ≥ 2	19 (67.9%)	7 (25%)

Fisher exact test: *p* = 0.003, there was also a steep fall-over in the number of patients with ECOG ≥ 2 points before and after the administration of alectinib

### CNS efficacy in patients with LM ± BM

Nine patients were diagnosed with LM (four patients with LM, five patients with LM + BM), of whom seven patients presented with typical clinical symptoms and linear enhancement in MRI at the time of diagnosis, one patient only had linear enhancement in MRI without CNS-related symptoms, while another one patient was found to have tumor cells in CSF without typical manifestation in MRI. All patients had uncontrolled CNS metastases before the initiation of alectinib. A complete 100% (8/8) of patients were reported to have significant improvement in CNS-related symptoms; furthermore, all patients (7/7) were no longer in need of mannitol or corticosteroids following the administration of alectinib, and seven patients experienced improvement in ECOG by at least one point (Tables 8 and 9). With a median follow-up of 16.8 months (95% CI: 4.1–28.7 m), CNS-TTP for patients with LM was 408d (Additional file 1: Fig. S1).

**Table 8:** Characteristics of patients with LM before the treatment of alectinib, *n* = 9

Evidence of LM diagnosis	Symptoms + MRI + CSF, *n* = 1 Symptoms + MRI, *n* = 7 MRI, *n* = 1
Accompanied with BM	*n* = 5
Previous history of ALK-TKI	ALK-TKI naive, *n* = 4 Crizotinib-resistant, *n* = 2 Other second generation ALK-TKIs, *n* = 3
Uncontrolled CNS metastases	*n* = 9
CNS-related symptoms	*n* = 8
ECOG	
0–1 ≥ 2	*n* = 1 *n* = 8
Number of patients needed mannitol or corticosteroids	*n* = 7

**Table 9:** Characteristics of patients with LM after the treatment of alectinib *n* = 9

CNS-related symptoms	*n* = 0
ECOG	
0–1 ≥ 2	*n* = 1 *n* = 8
Number of patients needed mannitol or corticosteroids	*n* = 0

### CNS-TTP between patients with symptomatic and asymptomatic BM in cohorts 1 and 2

**Fig. 4:**
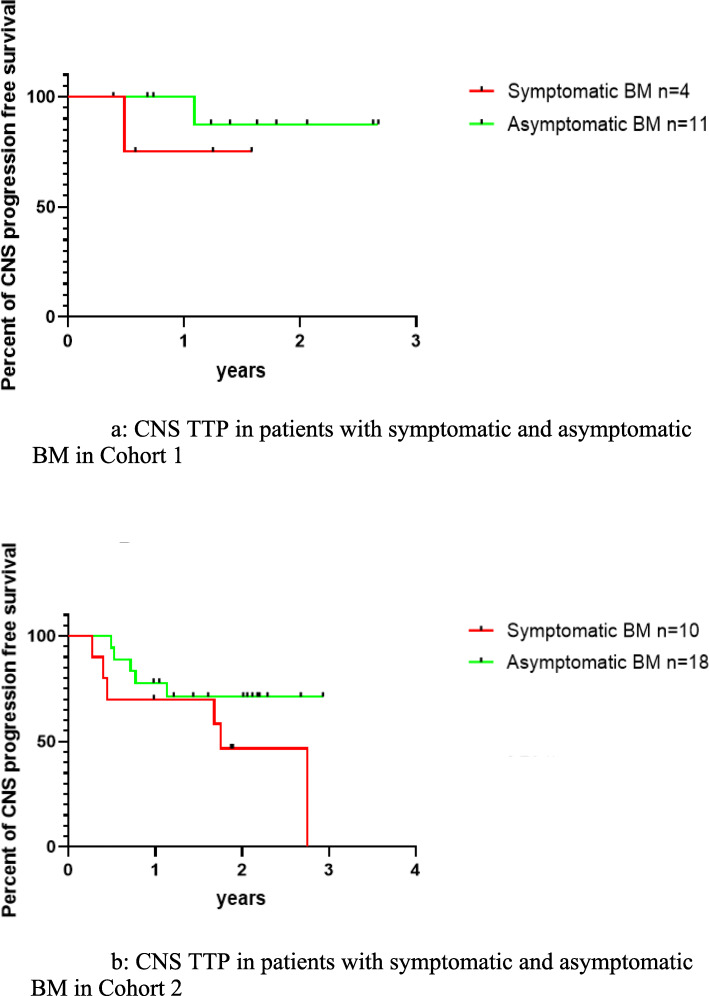
**a** CNS TTP in patients with symptomatic and asymptomatic BM in cohort 1. CNS TTP for patients with symptomatic BM and patients with asymptomatic BM in cohort 1was NE vs NE, *p* = 0.394, HR = 3.1(95% CI: 0.12 to 79.0). **b** CNS TTP in patients with symptomatic and asymptomatic BM in cohort 2. CNS TTP for patients with symptomatic BM and patients with asymptomatic BM in cohort 2 was 21.5 m vs NE, *p* = 0.168, HR = 2.24 (95% CI: 0.65 to 7.7)

In this part of the analysis, patients with LM and patients with controlled BM were excluded (cohort 1: *n* = 15, cohort 2: *n* = 28). In cohort 1, poor performance status (ECOG ≥ 2: 50% vs 0%, *p* = 0.057), measurable CNS lesions (100% vs 36.4%, *p* = 0.077), and multiple BM (≥ 4: (100% vs 27.3%, *p* = 0.026) were more often seen in patients with symptomatic BM (Additional file 2: Table S1a). No statistically significant difference was showed in CNS-TTP between patients with symptomatic and asymptomatic BM in cohort 1 (*p* = 0.394, HR = 3.1, 95% CI: 0.12–79.0) (Fig. 4a). In cohort 2, baseline characteristics were also described in Additional file 2: Table S1b; similarly, patients with symptomatic BM were found to have poor performance status (ECOG ≥ 2: 50% vs 11.1%, *p* = 0.063), measurable CNS lesions (70% vs 44.4%, *p* = 0.254), and multiple BM (90% vs 38.9%, *p* = 0.016) more frequently. Likewise, there was also no statistically significant difference in CNS-TTP between patients with symptomatic and asymptomatic BM in cohort 2 (*p* = 0.168, HR = 2.24, 95% CI: 0.65–7.7) (Fig. 4b).

### CNS-TTP between patients with different numbers of BM in cohorts 1 and 2

In this part of the analysis, patients with LM and patients with controlled BM were excluded. Included patients were further categorized into two groups (≥ 4 BM vs 1–3 BM) in cohorts 1 and 2. No statistically significant difference was demonstrated in CNS-TTP between patients with different numbers of BM (cohort 1: NE vs NE, *p* = 0.0925, HR undefined; cohort 2: 33.5 m vs NE, *p* = 0.316, HR = 1.9, 95% CI: 0.58–6.4) (Additional file 1: Fig. S2).

### Extracranial and overall efficacy

The ex-ORR was 83.3%, 24.1%, and 33.3% in patients with or without extracranial target lesions and nearly all patients achieved disease control in extracranial lesions, with only one patient in cohort 2 evaluated as PD. For patients with extracranial target lesions, 90.9%, 50%, and 66.7% of patients demonstrated response. Taken together, o-ORR was found to be 70%, 53.1%, and 30.8% in each cohort (Additional file 2: Table S2). At the time of data cutoff, in these three cohorts, 5, 17, and 6 patients were reported to develop progression events. In cohort 1, three patients experienced intracranial progression, while another two patients had extracranial progression; in cohort 2, eight patients developed progression only in CNS, while six patients experienced extracranial progression, and three patients had intracranial and extracranial progression simultaneously; in cohort 3, two patients experienced CNS progression while four patients were reported to have extracranial progression; intracranial oligo-progression was the main progression pattern for patients who developed CNS progression (11/14). No patient died in cohort 1 while 5 death events related to tumor progression or complications were confirmed in cohort 2; in cohort 3, one patient committed suicide and the other died of myocardial infarction without evidence of a progression event, while another death event was relevant to tumor progression (Additional file 2: Table S3). PFS and OS in the three cohorts are described in Additional file 1: Fig. S3.

## Discussion

Patients diagnosed with advanced ALK+NSCLC are more prone to develop CNS metastases [3–6] compared with those patients without driver gene mutation. Crizotinib had been reported to show dismal intracranial efficacy [15, 16]; hence, CNS is a common progression site following the treatment of first-generation ALK-TKI [3, 5, 6]. Therefore, second-generation ALK-TKIs with improved CNS activity had been developed to generate better CNS-protective effects [8, 9, 17–21]. Alectinib with high penetration rate across the BBB had been substantiated with potent intracranial efficacy in several clinical trials both in first-line and crizotinib-resistant settings [8, 9]. However, patients with symptomatic or unstable CNS metastases were excluded in all clinical trials of alectinib [4–6, 10, 11]; until now, mainstream strategies in clinical practice for these patients have been SRS, WBRT, and surgery, which probably lead to some neurological complications. Some researchers have even suggested that patients with ALK+NSCLC were particularly prone to develop RN [13, 14]. Therefore, efforts are urgently needed to investigate whether alectinib can also demonstrate robust CNS activity in patients with symptomatic CNS metastases so as to delay or reduce the need for local treatment. Additionally, there have been limited data on alectinib in patients resistant to other second-generation ALK-TKIs.

Intracranial efficacy of alectinib in ALK-TKI naive and crizotinib-resistant patients from our study was consistent with previous findings. Moreover, alectinib also demonstrated robust CNS activity for patients who develop progression only in CNS following the treatment of other second-generation ALK-TKIs. Moreover, most patients with symptomatic BM/LM experienced significant alleviation in CNS-related symptoms. As a whole, our results substantiated a potent CNS efficacy of alectinib in real-world settings.

A previous study from Lin et al. [22] had shown the robust CNS activity of alectinib in patients with symptomatic or large (≥ 1 cm) BM; however, patients in their study were not specifically classified based on the prior treatment of ALK-TKI. Our research presented more direct and elaborate results because we categorized patients into three cohorts according to their treatment history. Ceritinib had also been investigated in patients with refractory or symptomatic CNS metastases in the ASCEND-7 study [23, 24]. In this study, patients were also specifically divided into several cohorts based on their prior treatment with crizotinib and brain radiotherapy. However, their results might be less compelling because improvement in CNS-related symptoms was not reported in this study. Furthermore, previous research revealed that alectinib had a higher penetration rate across the BBB compared with other second-generation ALK-TKIs such as ceritinib [7]. Our results also indicated that alectinib could produce further inhibition in CNS lesions following treatment with other second-generation ALK inhibitors because most patients in cohort 3 presented reasonably good response.

Furthermore, to the best of our knowledge, we were first to report the CNS efficacy of alectinib in patients with LM. Although only a small sample size of patients with LM was included, promising results from our research would still have a positive impact in clinical practice. We also elaborately described the treatment outcomes in patients with symptomatic BM/LM who were excluded in clinical trials of alectinib. We observed that most of them experienced significant improvement in CNS-related symptoms. Additionally, CNS efficacy of alectinib between patients with symptomatic or asymptomatic BM was compared in our research. Our results showed that patients with symptomatic or asymptomatic BM could comparatively benefit from alectinib because there was no statistically significant difference in CNS-TTP between these two groups. Therefore, based on our findings, it might be reasonable for clinicians to defer the timing of RT for patients with symptomatic CNS lesions.

Our research had many limitations, and several questions were still not resolved. Our study was a retrospective analysis with a relatively small sample size; hence, our results must be treated with great caution. Besides, symptom relief, which was based on patients’ subjective reports rather than quantitative questionnaires, could not be recorded objectively and accurately, which might give rise to less accurate results. In addition, tumor burden of CNS metastases might be underestimated because the definition and evaluation of intracranial lesions from our research were based on RECIST 1.1 rather than mRECIST 1.1. However, RECIST 1.1, as one kind of evaluation criterion taking intracranial and extracranial lesions together, might be more practical and convenient in real-world settings. Moreover, although our study demonstrated promising efficacy of alectinib for patients with LM, it should be noted that a small sample size of patients was included. Hence, more data are needed to substantiate the long-term benefits of alectinib for LM. In addition, patients’ follow-up could not be performed uniformly; thus, parameters reflecting short-term efficacy could not be calculated accurately.

Last but not least, optimal timing of RT is still in need of further investigation. When referring to the value and optimal timing of RT, BM and LM should be analyzed separately. For patients with LM, they enjoyed rather dismal prognosis (overall survival 3–6 months) in the era of chemotherapy. Because most cases of LM manifest as disseminated lesions in meninges, many scholars once explored whether WBRT could improve the prognosis for these patients; unfortunately, prior studies showed limited improvement in CNS response and no survival benefit of WBRT [7, 25]. Since we stepped into the era of targeted therapy a decade ago, to date, there have been more treatment options for LM. Several researchers reported that patients with LM treated with osimertinib could live approximately 15 months [26–28]. Our results also demonstrated favorable efficacy of alectinib in LM. Therefore, TKIs with robust intracranial activity should be deemed as the vital options for LM, although more data are needed. At present, RT is more commonly used for alleviating symptoms in patients with bulky disease; meanwhile, it could also act as a salvage therapy when TKIs fail.

As for patients with BM, it has been widely accepted that patients with EGFR/ALK-positive NSCLC are more prone to develop BM, for whom repeated interventions for CNS lesions are highly common [29]. There is no doubt that with the help of sequential therapy of multiple generations of TKIs and local treatment, patients diagnosed with EGFR/ALK-positive NSCLC with BM can live significantly longer than before. Up to now, optimal timing of RT for these patients has always been a hot topic. Previous studies indicated that RT plus TKI showed some short-term benefits compared with TKI alone. For example, Chen et al. found that combination strategy could improve CNS progression-free survival for EGFR-mutated NSCLC [30]; other scholars reported that patients who received RT before crizotinib experienced longer PFS than those without [31]; results from the ALEX study also suggested that patients with prior RT demonstrated numerically higher CNS response rate and numerically lower risk in intracranial progression [9]. However, inconsistent conclusions were reached in terms of long-term benefits for combination strategy, Magnuson et al. found that patients who received upfront SRS followed by EGFR-TKI presented superior PFS and OS compared with those who received TKI followed by SRS or WBRT at intracranial progression [32]. Conversely, research from Chen et al. and Jiang et al. failed to show survival benefits of upfront RT (SRS or WBRT) [30, 33].

It should be noted that the aforementioned studies had several limitations; for example, TKIs with potent CNS efficacy were inaccessible in some studies. Moreover, some researchers failed to classify patients and the technique of RT more specifically. Actually, there have been two main kinds of classifications for patients with BM in clinical practice.

First, we usually categorize patients according to their symptoms. Many clinicians prefer to conduct local treatment for patients with symptomatic BM so as to alleviate CNS-related symptoms as soon as possible and prolong the duration of disease control. Our results indicated that most patients with symptomatic CNS metastases experienced significant alleviation in symptoms when treated with alectinib alone; meanwhile, although patients with symptomatic BM had larger and more CNS lesions, they still demonstrated similar CNS-TTP compared with asymptomatic patients. Based on these data, alectinib might defer or lower the need of local treatment for patients with symptomatic BM.

Second, patients can also be classified based on the number of BM. In clinical practice, patients with oligo-BM (1-3 or 1-5 BM) are eligible for SRS, while WBRT is usually applied to patients with multiple BM. Recent research revealed that upfront SRS could bring survival benefits for patients with oligo-BM in the era of osimertinib; however, prescribing WBRT in advance failed to demonstrated such advantages [34, 35]. Nonetheless these studies were mainly focused on patients with EGFR-mutated NSCLC, whereas no related research has been reported in ALK+ NSCLC. As more TKIs with robust CNS activity become accessible to patients with ALK+ NSCLC, therefore, whether upfront SRS could also demonstrate long-term benefits in this situation merits further exploration. In addition, previous findings suggested that most patients with baseline BM would develop intracranial multi-progression following treatment with crizotinib [31]; hence, some scholars harbor the idea that patients with BM might lose the chance of SRS at the time of intracranial progression. Conversely, our results indicated that intracranial oligo-progression was much more common in patients who developed CNS progression, which could be possibly explained by the favorable CNS-protective effect of alectinib and closed MRI follow-up to detect early progression. Therefore, patients who received alectinib might still have the chance of SRS at intracranial progression.

In addition, given the increasing attention to QoL in ALK+ patients who had fairly long survival, functional PFS or symptom-free survival rather than intracranial PFS or overall survival might be more meaningful primary endpoints in future [36].

## Conclusions

Our research substantiated the potent CNS efficacy of alectinib in real-world settings. Most patients with symptomatic CNS metastases experienced significant alleviation in symptoms; moreover, our results suggested that patients with symptomatic or asymptomatic BM could comparatively benefit from alectinib because there was no statistically significant difference in CNS-TTP between these two groups. Therefore, based on our findings, alectinib might defer or lower the need of local treatment for patients with symptomatic CNS metastases. However, our conclusions should be treated cautiously owing to our limited sample size.

## Supplementary Information

**Additional file 1: Figures S1-S3**. **Figure S1**—CNS TTP for patients with LM. **Figure S2a**—CNS TTP for patients with BM 1-3 vs ≥4 in Cohort 1. **Figure S2b**—CNS TTP for patients with BM 1-3 vs ≥4 in Cohort 2. **Figure S3a**—progression free survival in three cohorts. **Figure S3b**—overall survival in three cohorts.

**Additional file 2: Table S1-S3**. **Table S1a**—baseline characteristics between patients with symptomatic and asymptomatic BM in Cohort 1. **Table S1b**—baseline characteristics between patients with symptomatic and asymptomatic BM in Cohort 2. **Table S2**—efficacy in extracranial lesions and overall efficacy. **Table S3**—progression pattern and survival outcome at the time of data cut-off.

## Data Availability

The datasets generated and analyzed during this study are available from the corresponding authors on reasonable request.

## Abbreviations

NSCLC: Non small-cell lung cancer
TKI: Tyrosine kinase inhibitors
ALK: Anaplastic lymphoma kinase
CNS: Central nervous system
BM: Brain metastases
LM: Leptomeningeal metastases
QoL: Quality of life
BBB: Blood-brain barrier
RN: Radio-necrosis
RT: Radiotherapy
SRS: Stereotactic radiosurgery
WBRT: Whole brain radiotherapy
RECIST: Response Evaluation Criteria in Solid Tumors version
ic-ORR: Intracranial objective response rate
ic-DCR: Intracranial disease control rate
ex-ORR: Extracranial objective response rate
ex-DCR: Extracranial disease control rate
o-ORR: Overall objective response rate
o-DCR: Overall disease control rate
CI: Confidence interval
CR: Complete response
PR: Partial response
ic-DOR: Intracranial duration of response
CNS TTP: CNS time to progression
PFS: Progression free survival
OS: Overall survival
