# Supplementary material for: Intracranial efficacy of alectinib in ALK-positive NSCLC patients with CNS metastases—a multicenter retrospective study
Source: BMC Med. 2022 Jan 18;20:12. doi: 10.1186/s12916-021-02207-x (PMC8764827; doi:10.1186/s12916-021-02207-x)
Supplement: Supplementary file 1 — Additional file 1: Figures S1-S3. Figure S1—CNS TTP for patients with LM. Figure S2a—CNS TTP for patients with BM 1-3 vs ≥4 in Cohort 1. Figure S2b—CNS TTP for patients with BM 1-3 vs ≥4 in Cohort 2. Figure S3a—progression free survival in three cohorts. Figure S3b—overall survival in three cohorts. [file 12916_2021_2207_MOESM1_ESM.pdf]

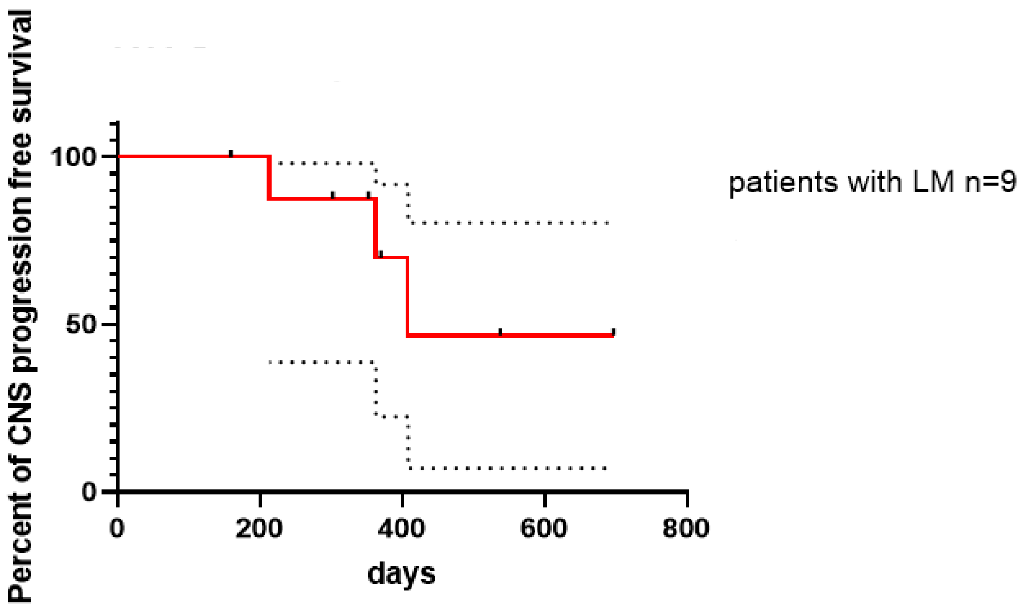

FigS1: CNS TTP for patients with LM

9 patients with LM, with median follow-up of 16.8 months, CNS TTP was 408d.

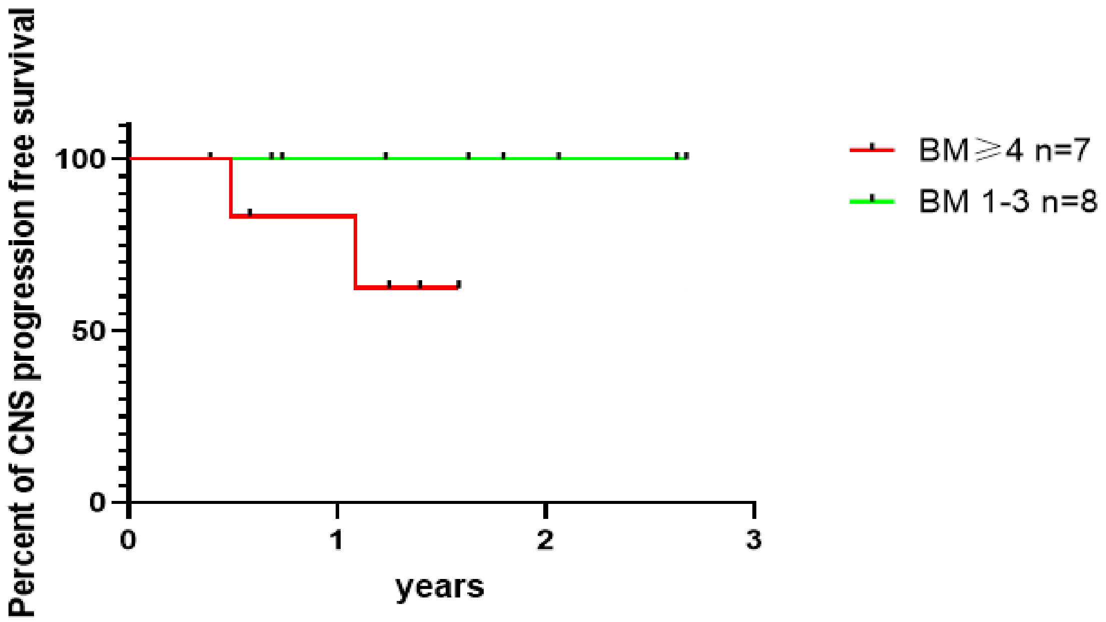

FigS2a: CNS TTP for patients with BM 1-3 vs ≥4 in Cohort 1

CNS TTP for patients with different number of BM in Cohort 1 was NE vs NE, p=0.0925, HR undefined.

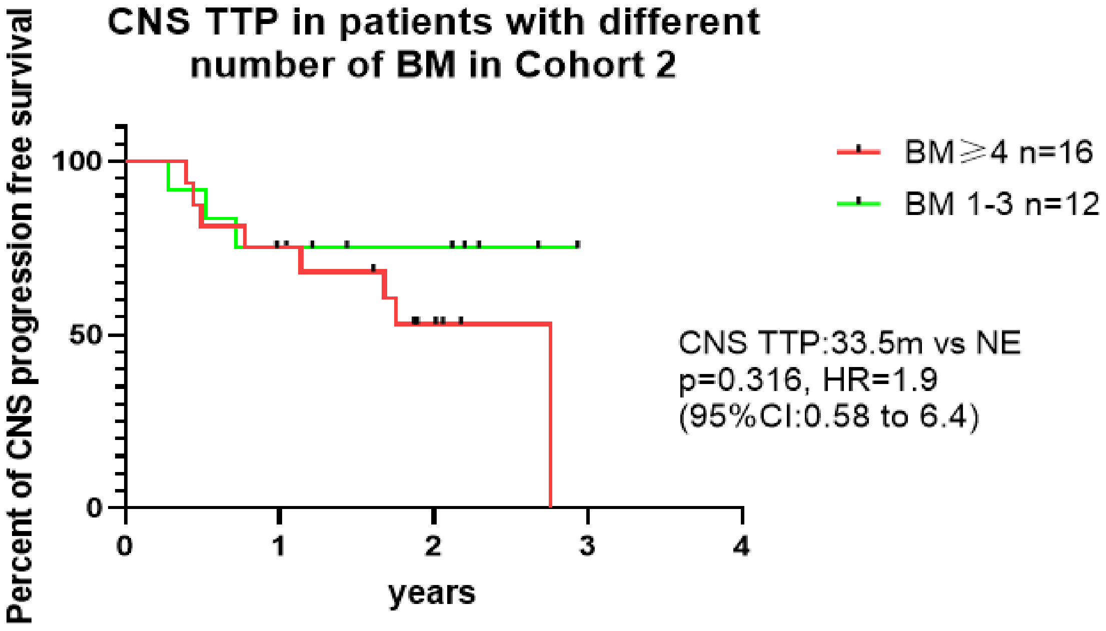

FigS2a: CNS TTP for patients with BM 1-3 vs ≥4 in Cohort 2

CNS TTP for patients with different number of BM in Cohort 2 was 33.5m vs NE, p=0.316, HR=1.9(95%CI: 0.58 to 6.4).

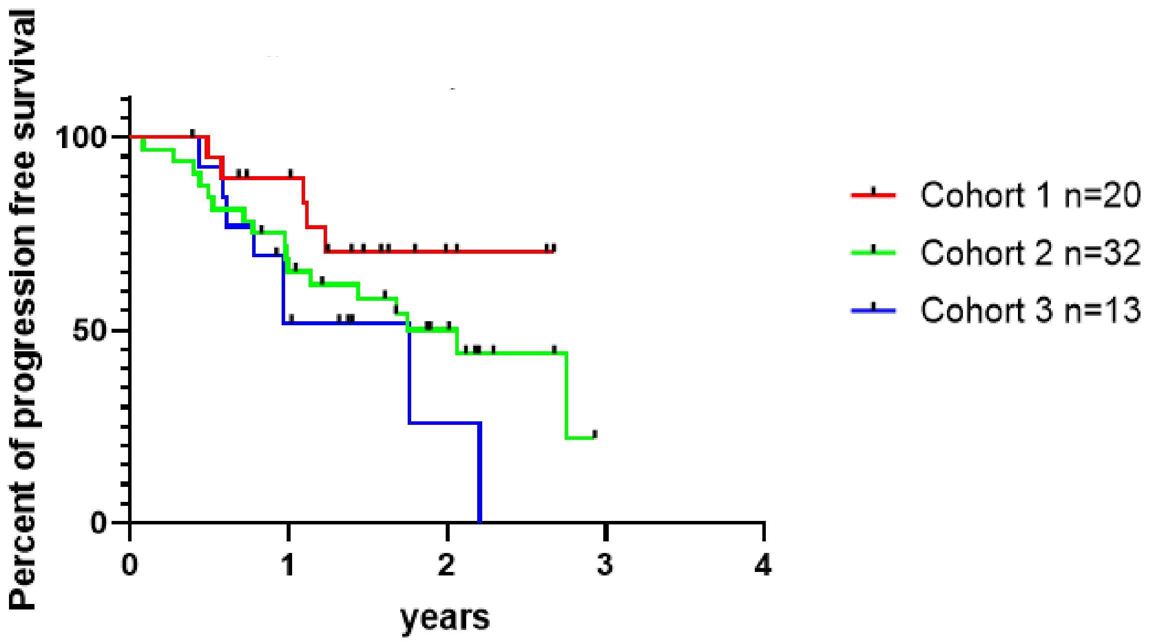

FigS3a: progression free survival in three cohorts

With median follow-up of 19.2 months, 22.5 months, 15.8 months in these three cohorts respectively, PFS was NE vs 24.7 months vs 21.2 months.

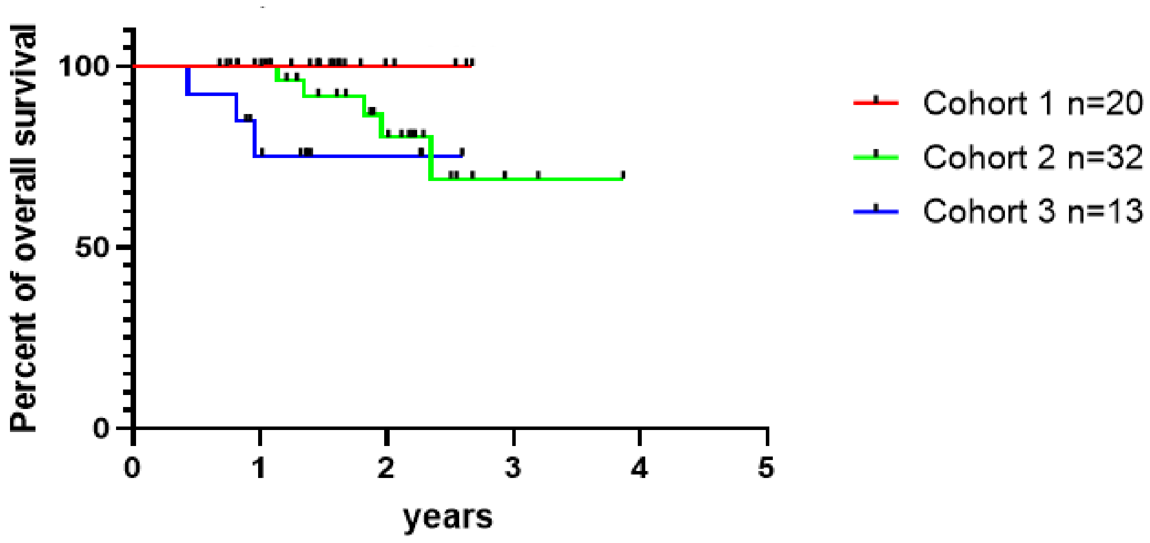

FigS3b: overall survival in three cohorts

With median follow-up of 19.2 months, 22.5 months, 15.8 months in these three cohorts respectively, OS was NE vs NE vs NE.
