# Supplementary material for: Intracranial efficacy of alectinib in ALK-positive NSCLC patients with CNS metastases—a multicenter retrospective study
Source: BMC Med. 2022 Jan 18;20:12. doi: 10.1186/s12916-021-02207-x (PMC8764827; doi:10.1186/s12916-021-02207-x)
Supplement: Supplementary file 2 — Additional file 2: Table S1-S3. Table S1a—baseline characteristics between patients with symptomatic and asymptomatic BM in Cohort 1. Table S1b—baseline characteristics between patients with symptomatic and asymptomatic BM in Cohort 2. Table S2—efficacy in extracranial lesions and overall efficacy. Table S3—progression pattern and survival outcome at the time of data cut-off. [file 12916_2021_2207_MOESM2_ESM.docx]

**TableS1a: baseline characteristics between patients with symptomatic and asymptomatic BM in Cohort 1**

| Cohort 1 | patients with symptomatic BM n=4 | patients with asymptomatic BM n=11 | P value |
| --- | --- | --- | --- |
| male  female | 0(0)  4(100%) | 4(36.4%)  7(63.6%) | 0.516 |
| age: ＜65  age: ≥65 | 4(100%)  0(0) | 10(90.9%)  1(9.1%) | 1 |
| ECOG: 0-1  ECOG: ≥2 | 2(50%)  2(50%) | 11(100%)  0 | 0.057 |
| never smoker  current or former smoker | 4(100%)  0(0) | 8(72.7%)  3(27.3%) | 0.516 |
| with CNS target lesions  without CNS target lesion | 4(100%)  0(0%) | 4(36.4%)  7(63.6%) | 0.077 |
| BM 1-3  BM≥4 | 0(0)  4(100%) | 8(72.7%)  3(27.3%) | 0.026 |

**Patients with LM and patients with controlled BM were excluded in this part of analysis**

**TableS1b: baseline characteristics between patients with symptomatic and asymptomatic BM in Cohort 2**

| Cohort 1 | patients with symptomatic BM n=10 | patients with asymptomatic BM n=18 | P value |
| --- | --- | --- | --- |
| male  female | 6(60%)  4(40%) | 9(50%)  9(50%) | 0.705 |
| age: ＜65  age: ≥65 | 9(90%)  1(10%) | 18(100%)  0(0) | 0.357 |
| ECOG: 0-1  ECOG: ≥2 | 5(50%)  5(50%) | 16(88.9%)  2(11.1%) | 0.063 |
| never smoker  current or former smoker | 6(60%)  4(40%) | 13(72.2%)  5(27.8%) | 0.677 |
| with CNS target lesions  without CNS target lesion | 7(70%)  3(30%) | 8(44.4%)  7(55.6%) | 0.254 |
| BM 1-3  BM≥4 | 1(10%)  9(90%) | 11(72.7%)  7(38.9%) | 0.016 |

**Patients with LM and patients with controlled BM were excluded in this part of analysis**

**TableS2: efficacy in extracranial lesions and overall efficacy**

|  | **Cohort 1 n=20** | **Cohort 2 n=32** | **Cohort 3 n=13** |
| --- | --- | --- | --- |
| extracranial ORR in patients with extracranial lesion | 83.3%  [95%CI:51.6%-97.9%]  (10/12) | 24.1%  [95%CI:10.3%-43.5%]  (7/29) | 33.3%  [95%CI:9.9%-65.1%]  (4/12) |
| extracranial ORR in patients with extracranial target lesion | 90.9%  [95%CI:58.7%-99.8%]  (10/11) | 50%  [95%CI:21.1%-78.9%]  (6/12) | 66.7%  [95%CI:22.3%-95.7%]  (4/6) |
| overall ORR in patients with or without target lesion | 70%  [95%CI:45.7%-88.1%]  (14/20) | 53.1%  [95%CI:34.7%-74.9%]  (17/32) | 30.8%  [95%CI:9.1%-61.4%]  (4/13) |

**TableS3: progression pattern and survival outcome at the time of data cut-off**

|  | **Cohort 1 n=20** | **Cohort2 n=32** | **Cohort3 n=13** |
| --- | --- | --- | --- |
| intracranial progression  extracranial progression  intracranial and extracranial progression | 3  2  0 | 8  6  3 | 2  4  0 |
| Intracranial oligo-progression | 2 | 8 | 1 |
| Intracranial multi-porgression | 0 | 3 | 0 |
| Death | 0 | 5 | 3  (1 patient committed suicide ,  1 patient died of myocardial infarction without the evidence of disease progression) |

**Pattern of intracranial progression was only recorded for patients without LM in the baseline**
